# Role of Carcinoembryonic Antigen in Severity Assessment and Mortality Prediction in COVID-19 Patients

**DOI:** 10.7759/cureus.69894

**Published:** 2024-09-22

**Authors:** Md. Ashraful Hassan Anjan, Quazi Mamtaz U Ahmed, Abdullah Al Masum, Chowdhury Adnan Sami, Md. Abdul Matin, Mohammad Syedul Islam, Fazle R Chowdhury, Shohael Mahmud Arafat, Mahbubur Rahman, Md Nazmul Hasan

**Affiliations:** 1 Internal Medicine, Bangabandhu Sheikh Mujib Medical University, Dhaka, BGD; 2 Internal Medicine, Nilphamari Sadar Hospital, Nilphamari, BGD

**Keywords:** biomarker comparison, carcinoembryonic antigen, carcinoembryonic antigen (cea), covid-19, mortality prediction, severity categorization

## Abstract

Background

Early risk stratification of COVID-19 may yield a better prognosis by tailoring effective treatment strategies. Recent studies have identified that elevated carcinoembryonic antigen (CEA) has prognostic value in terms of disease severity and mortality in patients with pneumonia. This study aims to explore the potential of CEA as a marker for both severity assessment and mortality prediction in COVID-19 patients.

Methods

From August 2020 to October 2021, we conducted this observational study in which patients who tested positive for COVID-19 by reverse transcription polymerase chain reaction (RT-PCR) or had high-resolution computed tomography (HRCT) chest suggestive of COVID-19 were included on day 0 of their admission to the COVID unit. Patients were classified into mild, moderate, severe, and critical according to the World Health Organization (WHO) guidelines. Blood samples were collected for complete blood count (CBC), C-reactive protein (CRP), ferritin, and CEA on days 0, 3, 7, and 14 of admission. The patient's profile was used to obtain lactate dehydrogenase (LDH), D-dimer, and HRCT scores [based on COVID-19 reporting and data system (CO-RADS) grade]. We used receiver operating characteristic (ROC) curves with Youden's index to find the initial (day 0) critical values of CEA for each of mild, moderate, severe, and critical COVID-19. The Kaplan-Meier survival curve was used to predict mortality with the best initial (day 0) cut-off value of CEA.

Results

Among 75 patients in this study, 15, 20, 19, and 21 were in the mild, moderate, severe, and critical groups, respectively; most were male (68%), and mortality was 18 (24%). Spearman's rank correlation test demonstrates a strong correlation between COVID-19 severity and changes in CEA. In the ROC curves, the area under the curve (AUC) value of CEA was higher among markers in all classifications except for mild to moderate disease. The AUC and critical values of CEA were as follows: for mild to moderate (0.948), 2.5 ng/ml; moderate to severe (1.000), 6.02 ng/ml; and severe to critical (0.769), 11.75 ng/ml. The survival curve shows the best initial cut-off values for mortality outcomes: CEA ≥7.15, CRP ≥81.52, ferritin ≥680.68, lymphocyte percentage ≤7.5, and neutrophil lymphocyte ratio ≥12.7.

Conclusions

The initial levels of CEA can serve as markers for severity assessment and mortality outcome prediction of COVID-19.

## Introduction

Coronavirus disease 2019 (COVID-19) is a respiratory-borne illness that causes respiratory tract infections ranging from mild to severe, with complications such as acute respiratory distress syndrome (ARDS), severe pneumonia, coagulopathy, sepsis, shock, and mortality [1]. A cytokine storm, a massive inflammatory response, leads to a quick escalation to severe disease [2]. Hence, early risk stratification would be beneficial in predicting severe disease; thus, clinical measures can be taken for reducing morbidity and mortality [3,4]. Several markers have been identified as being related to a poor prognosis in the initial stage of COVID-19. These markers include C-reactive protein (CRP), interleukin-6 (IL-6), and D-dimer [1].

Carcinoembryonic antigen (CEA) is produced primarily in the gastrointestinal tissue of the developing fetus but then ceases. Numerous physiological and pathological processes have been linked to this mammalian membrane glycosylphosphatidylinositol (GPI)-anchored protein. For example, CEA is a member of the CEA-related cell adhesion molecules family (CEACAM6), discovered 50 years ago [5]. In addition, certain cancers, such as colorectal cancer (particularly with liver metastases), gastric cancer, and non-cancerous diseases, such as smoking, cirrhosis, chronic hepatitis, and pneumonia, have high levels of CEA [6,7]. In reaction to inflammation, type II pneumocytes and bronchiolar cells express higher levels of CEA in the respiratory system [8]. It has been shown that serum CEA can be used in severity and mortality prediction in patients with pneumocystis carinii pneumonia (PCP) infection with HIV [9,10]. One study showed that CEA increased in patients with complicated PCP pneumonia who developed ARDS, and very high CEA levels were associated with mortality in those patients [9]. 

An abundance of angiotensin-converting enzyme 2 (ACE2) receptors is present in different cells of bronchial lining epithelial cells, which serves as entry receptors of severe acute respiratory syndrome coronavirus 2 (SARS-CoV-2), making them a prime target for COVID [11,12]. Several autopsy investigations have shown that COVID-19 patients have a significant proliferation of interstitial fibroblasts and hyperplasia of type II pneumocytes [13]. Because type II pneumocytes are the progenitors in alveoli, infection with SARS-CoV-2 causes a considerable amount of alveolar epithelial cell death. It is followed by an abnormal regeneration of type II pneumocytes, increasing the synthesis of CEA and other repairing mechanisms [14].

Only a few studies have been conducted worldwide to investigate the correlation between the CEA and disease severity in COVID-19 patients, where they found that CEA level correlated with severe COVID-19 disease and initial high CEA has been associated with mortality [15]. However, the ideal CEA cut-off value to forecast disease severity has not yet been established. This study aims to assess the correlation between the initial value of CEA and disease severity and to predict mortality outcome prediction in COVID-19 patients. In addition, we compared CEA with hematological, biochemical, and radiological markers in terms of disease severity and mortality.

## Materials and methods

Study design and participants

From August 2020 to October 2021, we conducted this observational study. We recruited any adult patients aged ≥18 years who tested positive for COVID-19 by reverse transcription polymerase chain reaction (RT-PCR) from nasal swab or whose high-resolution computed tomography (HRCT) chest was suggestive of COVID-19 disease. Any patient who was a regular smoker or diagnosed with any of the following conditions were excluded from the study: malignant tumors (colorectal cancer, particularly with liver metastasis; gastric cancer; lung cancer; breast cancer; mucinous cancer of the ovary), inflammatory bowel disease, liver cirrhosis, pancreatitis, COPD, and diffuse parenchymal lung disease. Additionally, those who had been vaccinated against COVID-19 were excluded from the study.

Data collection

After recruitment on day 0, patients were classified initially as severe and non-severe by the preformed checklist according to the World Health Organization (WHO) [16]. Participants were followed till day 14; thereafter, the final classification of mild, moderate, severe, and critical cases was done according to the WHO. Blood samples were collected for complete blood count (CBC), CRP, serum ferritin, and CEA on days 0, 3, 7, and 14 of admission. From CBC, the lymphocyte percentage and neutrophil lymphocyte ratio (NLR) were derived. If any patient was discharged earlier than 14 days, we followed up the patient on an outpatient basis on days 7 and 14, whichever the case. As per hospital protocol, there was no provision for the admission of mild patients. Therefore, mild patients were enrolled on an outpatient basis from the triage of the COVID unit and followed up on days 7 and 14. Blood samples were collected on days 0 and 7 for mild cases; blood samples were not taken on days 3 and 14 for these patients. Lactate dehydrogenase (LDH), D-dimer, and HRCT chest score reports were collected from the patient's medical record. HRCT score was based on COVID-19 reporting and data system (CO-RADS) grade.

Laboratory details

The CEA assay was used to quantitatively measure carcinoembryonic antigen in human serum using the Atellica™ IM CEA Analyzer (Siemens Healthineers, Erlangen Germany). In addition, Atellica™ IM Ferritin assay for serum ferritin and Atellica™ CH High Sensitivity CRP assay for CRP were used (Siemens Healthineers). CBC was done using Sysmex XN-2000, an automated six-part differential hematology analyzer (Sysmex, Singapore)

Statistical analysis

Statistical analysis was performed using Microsoft Excel, IBM SPSS Statistics for Windows, Version 26.0 (Released 2019; IBM Corp., Armonk, NY, USA), and GraphPad Prism 7.0, Released April 2016 (Dotmatics, Boston, MA) . Continuous variables were shown as median+interquartile range (IQR), and categorical variables were expressed as percentages. A p-value <0.05% was considered significant. Chi-square test, one-way analysis of variance (ANOVA) with post hoc Tukey Honest Significant Difference (HSD) test and Bonferroni correction, repeated measures one-way ANOVA test, receiver operator characteristic (ROC) curve, Youden's J statistics, Spearman's rank correlation coefficient test, and Kaplan-Meier survival curve were applied for the analyses of qualitative and quantitative data. 

## Results

Sociodemographic characteristics

Among 75 patients in this study, 15 were in mild, 20 in moderate, 19 in severe, and 21 in critical groups. Table 1 shows that most patients were male (68%, 51) compared to female (32%, 24). Fifty percent of participants were more than 60 years of age, and patients with severe disease had a higher median age (p<0.05). The total in-hospital mortality was 18 (24%). Regarding body mass index (BMI), the distribution across different severity was not statistically significant (p=0.16). Among the participants, 73% of patients had comorbidities; 49% had multiple risk factors, 24% had a single risk factor, 59% had hypertension (HTN), 52% had diabetes mellitus (DM), 15% had ischemic heart disease (IHD), 13% had asthma, 9% had chronic kidney disease (CKD), and 3% had cerebrovascular disease (CVD). The severity of patients was associated with the presence of comorbidities (p<0.05). Of the total 75 patients, 4% were asymptomatic, and others presented with fever (80%), cough (80%), dyspnea (69%), and anorexia (68%). A considerable number of patients presented with anosmia (61%), loss of taste (61%), running nose (49%), myalgia (47%), and sore throat (27%) (Table 1).

**Table 1:** Baseline characteristics and clinical profile of the participants. BMI: body mass index; HTN: hypertension; DM: diabetes mellitus; IHD: ischemic heart disease; CKD: chronic kidney disease; CVD: cerebrovascular disease. *Chi-square test was applied to identify the level of significance.

Characteristics	Total Participants (n=75) Age (median, Q1-Q3), n (%)	Mild COVID (n=15) Median (Q1-Q3), n (%)	Moderate COVID (n=20) Median (Q1-Q3), n (%)	Severe COVID (n=19) Median (Q1-Q3), n (%)	Critical COVID (n=21) Median (Q1-Q3), n (%)	p value*
Age (years)	60 (47-68)	42 (36.5-51.5)	54.5 (48.5-62.75)	65 (51-70)	65 (60-70)	<0.05
Gender						
Male	51 (68%)	9 (12%)	15 (20%)	13 (17%)	14 (19%)	
Female	24 (32%)	6 (8%)	5 (7%)	6 (8%)	7 (9%)	
BMI						0.16
Normal	25 (33%)	8 (10%)	7 (9%)	4 (6%)	6 (8%)	
Overweight	27 (36%)	5 (7%)	6 (8%)	8 (10%)	8 (10%)	
Obese	23 (31%)	2 (4%)	7 (9%)	7 (9%)	7 (9%)	
Co-morbidities						<0.05
HTN	44 (59%)	5 (7%)	12 (16%)	14 (19%)	13 (17%)	
DM	39 (52%)	3 (4%)	12 (16%)	12 (16%)	12 (16%)	
IHD	11 (15%)	1 (1%)	3 (4%)	3 (4%)	4 (6%)	
Asthma	10 (13%)	0	6 (8%)	1 (1%)	3 (3%)	
CKD	7 (9%)	1 (1%)	0	2 (3%)	4 (6%)	
CVD	2 (3%)	0	0	1 (1.5%)	1 (1.5%)	
Symptoms						
Asymptomatic	3 (4%)	3 (4%)	0	0	0	
Fever	60 (80%)	9 (11%)	17 (23%)	17 (23%)	17 (23%)	
Cough	60 (80%)	11 (14%)	18 (24%)	17 (23%)	14 (19%)	
Dyspnea	42 (69%)		13 (17%)	18 (24%)	21 (28%)	
Sore throat	20 (27%)	7 (9%)	6 (8%)	7 (9%)	0	
Runny nose	37 (49%)	10 (13%)	7 (9%)	6 (8%)	14 (19%)	
Myalgia	35 (47%)	8 (11%)	16 (21%)	9 (12%)	2 (3%)	
Headache	16 (21%)	4 (5%)	6 (8%)	2 (3%)	4 (5%)	
Nausea/vomiting	1 (1%)	1 (1%)	0	0	0	
Diarrhea	-	0	0	0	0	
Abdominal pain	5 (7%)	0	0	3 (4%)	2 (3%)	
Rash	-	0	0	0	0	
Anosmia	46 (61%)	8 (11%)	12 (16%)	14 (19%)	12 (16%)	
Loss of taste	50 (66%)	8 (11%)	12 (16%)	14 (19%)	16 (20%)	
Anorexia	47 (63%)	4 (5%)	17 (23%)	14 (19%)	12 (16%)	

Table 2 shows correlation coefficient between severity and gender (rs=-0.016), severity and age (rs=0.530), between severity and BMI (rs=0.187), and severity and comorbidities (rs=0.303). This signifies moderate positive correlation between severity and age, weak positive correlation between severity and comorbidities, and very weak correlation between severity and both gender and BMI.

**Table 2:** Correlation between disease severity, gender, age, BMI, and comorbidities. BMI: body mass index.

		Gender	Age	BMI	Comorbidities
Severity	Correlation coefficient (rs)	-0.016	0.530	0.187	0.303
	Significance (two-tailed)	0.894	0.0001	0.108	0.008

Comparison of initial (D0) CEA, NLR, lymphocyte percentage, serum ferritin, LDH, CRP, D-dimer, and HRCT chest scores among different severities of COVID-19 patients

At day 0, the median+IQR of CEA was as follows: mild 1.53 (0.79-1.73), moderate 3.84 (2.46-4.44), severe 10.38 (7.1-13.72), and critical 17.67 (10.0-29.05). Table 3 shows a significant positive correlation between day 0 values of CEA, LDH, NLR, CRP, D-dimer, ferritin, and HRCT score with disease severity, whereas the lymphocyte percentage shows a significant negative correlation.

**Table 3:** Correlation between disease severity and day 0 values of markers (CEA, ferritin, CRP, lymphocyte percentage, NLR, LDH, D-dimer, and HRCT score). CEA: carcinoembryonic antigen; LDH: lactate dehydrogenase; CRP: C-reactive protein; NLR: neutrophil-lymphocyte ratio; HRCT: high-resolution computed tomography. *Non-severe vs. severe. ** Spearman's rank correlation coefficient test was applied.

	Variables	Correlation coefficient (rs)**	p value
Disease severity*	CEA	0.833	0.0001
	LDH	0.746	0.0001
	NLR	0.643	0.0001
	Lymphocyte %	-0.635	0.0001
	Ferritin	0.472	0.0001
	CRP	0.419	0.0001
	D-dimer	0.603	0.0001
	HRCT score	0.566	0.0001

**Figure 1:**
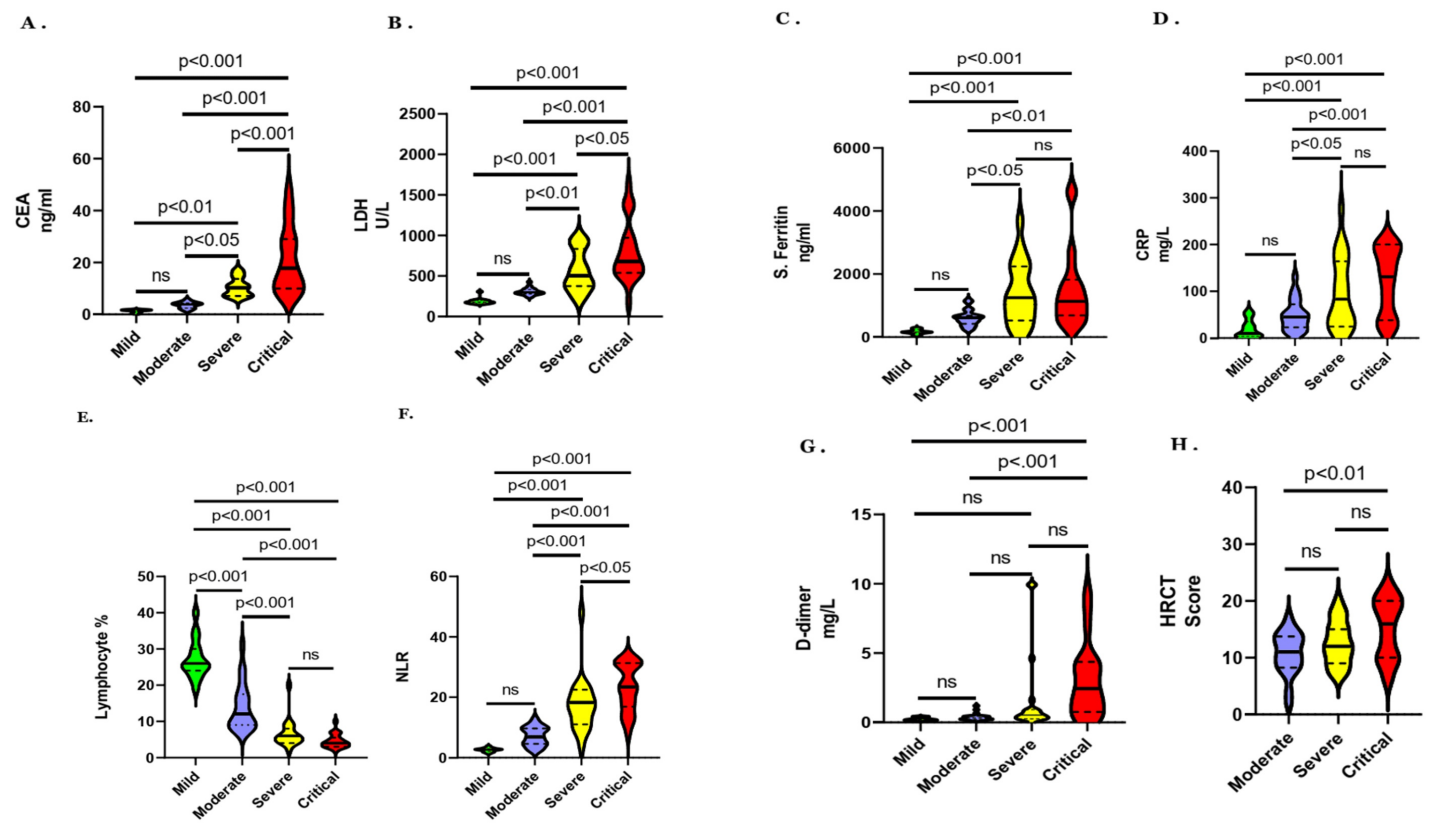
Comparison of CEA (A), LDH (B), ferritin (C), CRP (D), lymphocyte percentage (E), NLR (F), D-dimer (G), and HRCT scores (H) among different severities of COVID-19 patients. CEA: carcinoembryonic antigen; LDH: lactate dehydrogenase; CRP: C-reactive protein; NLR: neutrophil-lymphocyte ratio; HRCT: high-resolution computed tomography.

To compare the initial maximum values of CEA, NLR, lymphocyte percentage, serum ferritin, LDH, CRP, D-dimer, and HRCT chest score, we performed one-way ANOVA. The HRCT score values were not available in the mild group. Figure 1A-H shows the comparison of median values of the markers and HRCT score among different severities that is mild, moderate, severe, and critical with their significance.

Figure 1A shows that the difference in CEA values between mild and moderate is not significant but significant among other severities. Figure 1B shows that the difference in LDH values between mild and moderate is not significant but significant among other severities. Figure 1C shows that the difference in ferritin values between mild and moderate and between severe and critical is not significant but significant among other severities. Figure 1D shows that the difference in CRP values between mild and moderate and between severe and critical is not significant but significant among other severities. Figure 1E shows that the difference in lymphocyte percentage values between severe and critical is not significant but significant among other severities. Figure 1F shows that the difference in NLR values between mild and moderate is not significant but significant among other severities. Figure 1G shows that the difference in D-dimer values between mild and critical and between moderate and critical is significant but not significant among other severities. Figure 1H shows the difference in HRCT values between moderate and critical is significant but not significant among other severities. 

Changes in CEA over the disease period

**Figure 2:**
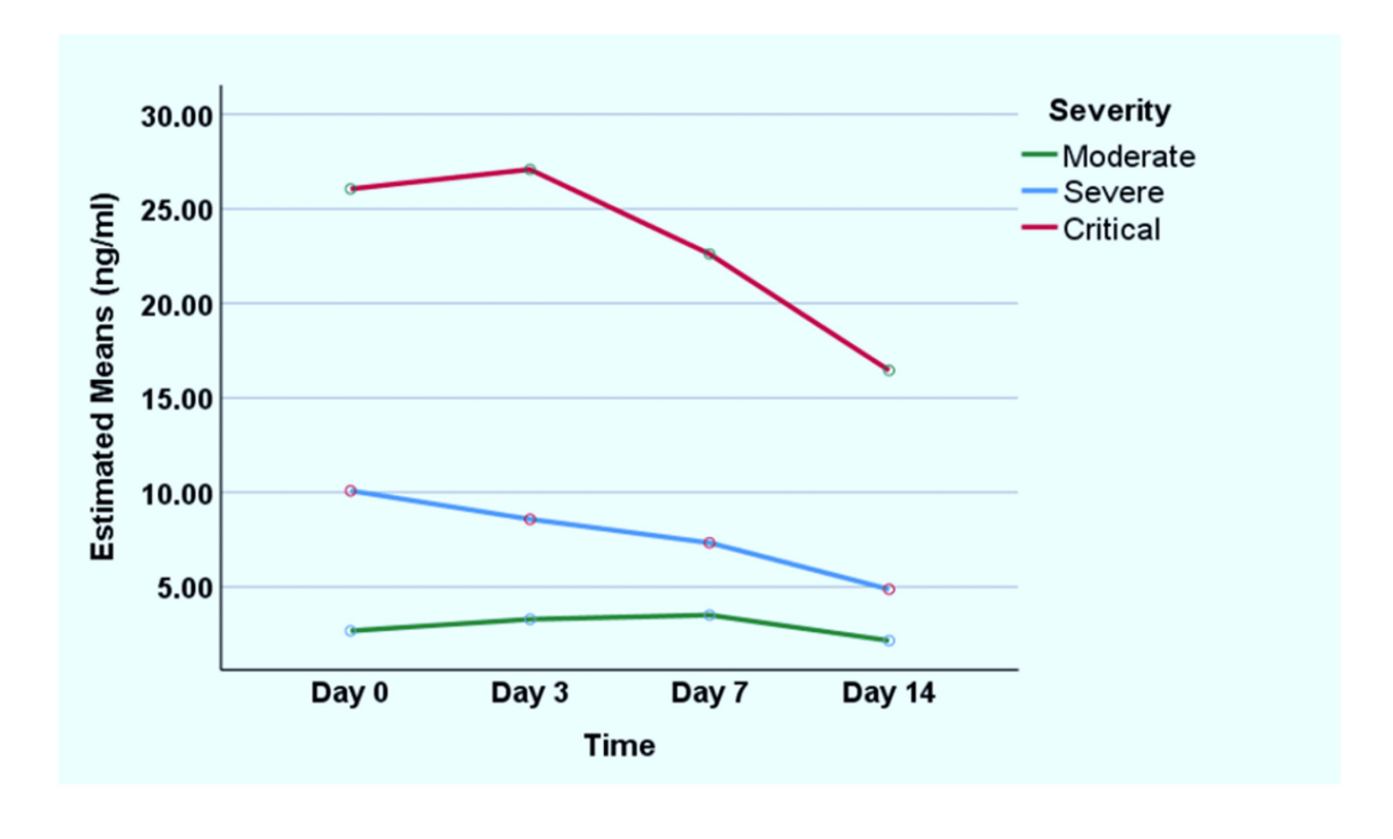
Transient rise of CEA. CEA: carcinoembryonic antigen.

A repeated measure one-way ANOVA was applied to analyze changes in CEA levels on days 0, 3, 7, and 14. Table 4 shows no significant mean differences among days 0, 3, and 7 (p>0.05). However, the standard differences are substantial between day 14 and other measures, that is, days 0, 3, and 7 (p<0.05), which signifies the transient rise of CEA initially during the disease period, which declines later. Figure 2 shows the changes in CEA levels over the disease period. 

**Table 4:** Pairwise comparisons of repeated measures of CEA. CEA: carcinoembryonic antigen. *Repeated measures one-way ANOVA test was done.

(I) Time	(J) Time	Mean difference (I-J)	p-value*	95% CI	
				Lower bound	Upper bound
Day_0	Day_3	0.439	1.000	-1.641	2.518
	Day_7	1.656	0.351	-0.760	4.071
	Day_14	4.275	0.0001	2.004	6.545
Day_3	Day_0	-0.439	1.000	-2.518	1.641
	Day_7	1.217	0.667	-0.920	3.354
	Day_14	3.836	0.003	1.160	6.511
Day_7	Day_0	-1.656	0.351	-4.071	0.760
	Day_3	-1.217	0.667	-3.354	0.920
	Day_14	2.619	0.004	0.722	4.517
Day_14	Day_0	-4.275	0.0001	-6.545	-2.004
	Day_3	-3.836	0.003	-6.511	-1.160
	Day_7	-2.619	0.004	-4.517	-0.722

Disease classification

**Figure 3:**
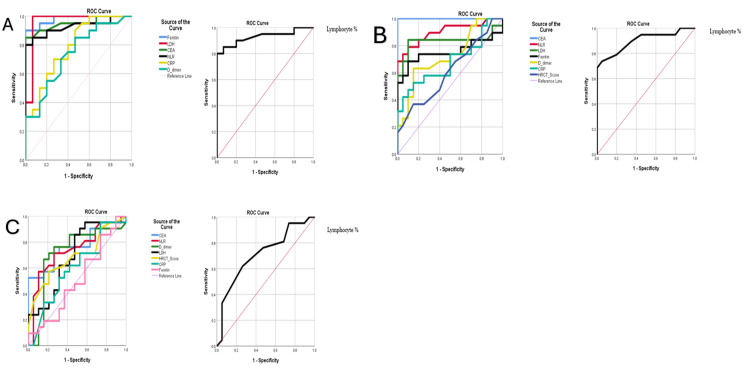
ROC curve: mild to moderate (A), moderate to severe (B), and severe to critical (C). ROC: receiver operating characteristic; CEA: carcinoembryonic antigen; LDH: lactate dehydrogenase; CRP: C-reactive protein; NLR: neutrophil-lymphocyte ratio; and HRCT: high-resolution computed tomography.

The ROC curves were applied to assess disease classification between (a) mild to moderate, (b) moderate to severe, and (c) severe to critical. (a) Mild to moderate: Figure 3A shows the ROC curves for CEA, NLR, lymphocyte percentage, ferritin, CRP, D-dimer, LDH, and HRCT scores to assess disease classification. The AUC values were ferritin (0.980), LDH (0.960), CEA (0.948), NLR (0.927), lymphocyte percentage (0.928), CRP (0.788), and D-dimer (0.737). The critical values were ferritin (316.75 ng/ml), LDH (223.5 U/L), CEA (2.5 ng/ml), NLR (4.14), lymphocyte percentage (18.5), CRP (11 mg/L), and D-dimer (0.2 mg/L) (Table 5). (b) Moderate to severe: Figure 3B shows the ROC curves for CEA, NLR, lymphocyte percentage, ferritin, CRP, D-dimer, LDH, and HRCT scores to assess disease classification. The AUC values were CEA (1.000), NLR (0.901), lymphocyte percentage (0.899), LDH (0.833), ferritin (0.755), D-dimer (0.726), CRP (0.682), and HRCT score (0.612). The critical values were CEA (6.02 ng/ml), NLR (10.88), lymphocyte percentage (7.5), LDH (353.5 U/L), ferritin (535.00 ng/ml), D-dimer (0.48 mg/L), CRP (82 mg/L), and HRCT score (14) (Table 5). (c) Severe to critical: Figure 3C shows the ROC curves for CEA, NLR, lymphocyte percentage, ferritin, CRP, D-dimer, LDH, and HRCT scores to assess disease classification. The AUC values were CEA (0.769), NLR (0.737), lymphocyte percentage (0.713), D-dimer (0.713), LDH (0.689), HRCT score (0.688), CRP (0.595), and ferritin (0.506). The critical values were CEA (11.75 ng/ml), NLR (18.55), lymphocyte percentage (4.5), D-dimer (1.65 mg/L), LDH (513.5 U/L), HRCT score (15), CRP (128.00 mg/Ld), and ferritin (886.4 ng/ml) (Table 5). 

**Table 5:** AUC and critical values. AUC: area under the curve; CEA: carcinoembryonic antigen; LDH: lactate dehydrogenase; CRP: C-reactive protein; NLR: neutrophil-lymphocyte ratio; HRCT: high-resolution computed tomography.

	Mild to moderate		Moderate to severe		Severe to critical		
Variables	AUC	Critical values	AUC	Critical values	AUC		Critical values
CEA	0.948	2.5 ng/ml	1.000	6.02 ng/ml	0.769		11.75 ng/ml
NLR	0.927	4.14	0.901	10.88	0.737		18.55
LDH	0.960	223.5 U/L	0.833	353.5 U/L	0.689		513.5 U/L
Lymphocyte percentage	0.928	18.5	0.899	7.5	0.713		4.5
Ferritin	0.980	316.75 ng/ml	0.755	535.00 ng/ml	0.506		886.4 ng/ml
D-dimer	0.737	0.2 mg/L	0.726	0.48 mg/L	0.713		1.65 mg/L
CRP	0.788	11.0 mg/L	0.682	82 mg/L	0.595		128.00 mg/L
HRCT			0.612	14	0.688	15	

Correlation between disease severity and dynamic changes of biochemical markers (CEA, ferritin, CRP) and hematological markers (lymphocyte percentage, NLR)

Table 6 describes the correlation coefficient between non-severe (moderate) versus severe (severe and critical) and changes of markers (CEA, ferritin, CRP, lymphocyte percentage, and NLR) on days 0, 3, 7, and 14. The correlation coefficients for CEA were as follows: day 0 (rs=0.833), day 3 (rs=0.799), day 7 (rs=0.793), and day 14 (rs=0.605). This comparison signifies a strong positive correlation between severity and CEA at day 0, which declines on days 3, 7, and 14. Between disease severity and NLR, we observed a moderate positive correlation, while a moderate negative correlation was noted between disease severity and lymphocyte percentage. However, a weak correlation was observed between severity and both ferritin and CRP.

**Table 6:** Correlation between disease severity and markers (CEA, ferritin, CRP, lymphocyte percentage, and NLR). CEA: carcinoembryonic antigen; CRP: C-reactive protein; NLR: neutrophil-lymphocyte ratio. *Non-severe (moderate) vs. severe (severe and critical). **Spearman's rank correlation coefficient test was applied.

	Variables	Correlation coefficient (rs)**			
		Day 0	Day 3	Day 7	Day 14
	CEA (rs)	0.833	0.799	0.793	0.605
	p value	0.0001	0.0001	0.0001	0.001
	NLR (rs)	0.643	0.587	0.760	0.606
	p value	0.0001	0.0001	0.0001	0.005
Disease severity*	Lymphocyte percentage (rs)	-0.635	-0.598	-0.762	-0.617
	p value	0.0001	0.0001	0.0001	0.004
	Ferritin (rs)	0.472	0.153	0.409	0.139
	p value	0.0001	0.295	0.001	0.517
	CRP (rs)	0.419	0.113	0.350	0.533
	p value	0.0001	0.434	0.007	0.006

Outcome of patients with specific initial (D0) cut-off points of CEA and other markers (CRP, ferritin, lymphocyte percentage, and NLR)

**Figure 4:**
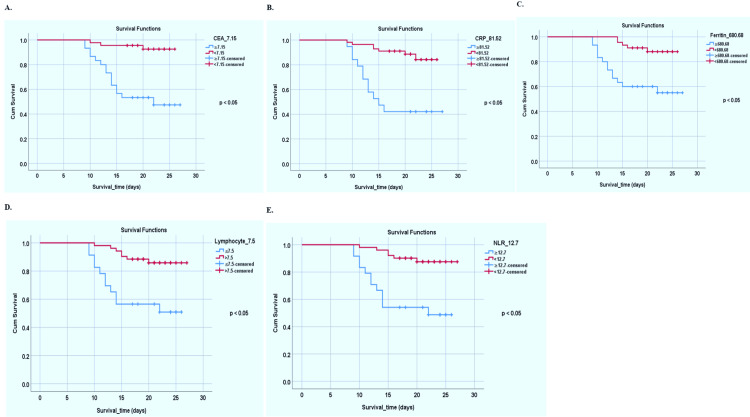
Kaplan-Meier curve with specific initial level (day 0) cut-off values of CEA (A), CRP (B), ferritin (C), lymphocyte percentage (D), and NLR (E). CEA: carcinoembryonic antigen; CRP: C-reactive protein; NLR: neutrophil-lymphocyte ratio.

CEA (ng/ml), CRP (mg/L), serum ferritin (ng/ml), lymphocyte percentage, and NLR measured on day 0 of hospital admission of the patient were used to analyze mortality using the Kaplan-Meier curve. Figure 4A-E shows the survival curves with these markers' best initial level cut-off values. The cut-off values are CEA ≥7.15, CRP ≥81.52, ferritin ≥680.68, lymphocyte percentage ≤7.5, and NLR ≥12.7. These values were obtained from Youden's J statistical analysis. Higher levels of these markers' initial level cut-off values had worse outcomes than patients with a lower level. A log rank (Mantel-Cox) test was applied for overall comparisons (p<0.05).

## Discussion

In this study, a total of 75 patients with COVID-19 were enrolled; among them, 68% were male. There were no significant gender differences among different severities like other studies (p>0.05) [15]. There was a significant, moderately positive correlation between age and disease severity. Also, the median age of patients was significantly different across different severity levels, similar to previous studies (p<0.05) [17]. Regarding BMI, we did not find any significant differences among different severity groups (p>0.05). In our study, 59% of patients had hypertension, 52% had diabetes, 15% had ischemic heart disease, 13% had asthma, 9% had CKD, and 3% had cerebrovascular disease. It was observed that the severity of patients was associated with comorbidities, like in the previous study [17]. Although cardiac disease and diabetes can cause a rise in CEA, the level of rise is minimum [18]. Also, in our study, we showed that CEA was raised for a transient period, and there was a decline over time (Figure 2), which suggested that it was due to the disease process and not due to comorbidities. In addition, DM patients were equally distributed among the moderate, severe, and critical groups of COVID-19.

There was no significant difference between mild and moderate patients in the median values of CEA, LDH, ferritin, CRP, D-dimer, and NLR, except in the lymphocyte percentage. However, significant differences were seen in the median values of CEA, LDH, ferritin, CRP, lymphocyte percentage, and NLR between those who were not severely affected (mild to moderate) and those who were severely affected (not critical). These findings support the previous three studies [19-21]. But in the case of D-dimer, it was not significant, which does not support the findings of the previous study [22], probably because it was not measured multiple times to see the changes. The findings of CEA values differentiating severe and critical patients differed from previous studies [15, 23]. Those studies showed no significant difference in CEA values between severe and critical. This may be because, in our study, CEA measurement was done multiple times, which was not done in previous studies.

In this study, the initial CEA (D0) level was more strongly correlated with disease severity than other markers. In addition, NLR and lymphocyte percentage were better correlated with disease severity than ferritin and CRP. In our study, a strong correlation was shown with LDH and disease activity, while D-dimer was moderately correlated; these findings were consistent with other previous studies [23].

The survival analysis was based on the D0 level with specific cut-off values of CEA (7.15 ng/ml), lymphocyte percentage (7.5%), NLR (12.7), ferritin (680.68 ng/ml), and CRP (81.52 mg/L) (p<0.05). Higher values of CEA, ferritin, and CRP associated with fatal outcomes were also observed in previous studies [15,23].

In our study, the difference in median values of HRCT scores between moderate and critical was significant (critical value 15, AUC (0.688)). The difference was insignificant between moderate and severe as well as severe and critical, and this is in line with the findings of other studies [24], where they showed a significant difference between non-severe (moderate) and severe as well as non-severe (moderate) and critical. There is a moderate positive correlation between disease severity and HRCT score (rs=0.566), as well as between HRCT score and CEA (rs=0.5930), as observed in other studies [19]. Finally, our study also showed the rise of CEA was transient due to the COVID-19 disease process, which is consistent with other studies [25].

Limitations

The study has specific limitations. Initially, it is a single-center study, which does not represent the entire population. The sample size was also small, which was mainly due to financial constraints. Furthermore, there exists a varied time interval between the appearance of symptoms and the patient's admission to the hospital or consultation, which can potentially impact the levels of parameters and the accurate representation of illness progression. Additionally, in accordance with the established procedure, this study did not include successive assessments of LDH, D-dimer, and repeat HRCT. Regarding successive changes in CEA over the disease period, mild cases were excluded; only moderate, severe, and critical patients were included (days 3, 7, and 14). Other inflammatory markers could not be evaluated due to financial constraints. It was also not possible to assess the persistent increase in CEA levels for more than 14 days in severe patients. Also, CEA level can be affected by cardiovascular disease or the presence of diabetes. As without comorbidities, there would be difficulty in recruiting patients; however, we tried to evenly distribute the patients with such comorbidities among all the groups.

## Conclusions

Patients with COVID-19 had higher initial serum CEA levels with increasing disease severity. In addition, higher initial CEA levels were associated with fatal outcomes. CEA can be used as a new indicator to forecast the severity and prognosis of COVID-19 and is comparable with other hematological, biochemical, and radiological markers.

## References

[1] Zhou P, Yang XL, Wang XG (2020). A pneumonia outbreak associated with a new coronavirus of probable bat origin. Nature.

[2] Soy M, Keser G, Atagündüz P, Tabak F, Atagündüz I, Kayhan S (2020). Cytokine storm in COVID-19: pathogenesis and overview of anti-inflammatory agents used in treatment. Clin Rheumatol.

[3] Guan WJ, Ni ZY, Hu Y (2020). Clinical characteristics of coronavirus disease 2019 in China. N Engl J Med.

[4] Thachil J, Tang N, Gando S (2020). ISTH interim guidance on recognition and management of coagulopathy in COVID-19. J Thromb Haemost.

[5] Tchoupa AK, Schuhmacher T, Hauck CR (2014). Signaling by epithelial members of the CEACAM family - mucosal docking sites for pathogenic bacteria. Cell Commun Signal.

[6] Asad-Ur-Rahman F, Saif MW (2016). Elevated level of serum carcinoembryonic antigen (CEA) and search for a malignancy: a case report. Cureus.

[7] M.S. Loewenstein, N. Zamcheck Carcinoembryonic antigen (CEA) levels in benign gastrointestinal disease states. Cancer.

[8] Abbona GC, Papotti M, Gugliotta P, Pecchio F, Rapellino M (1993). Immunohistochemical detection of carcinoembryonic antigen (CEA) in non-neoplastic lung disease. Int J Biol Markers.

[9] Bédos JP, Hignette C, Lucet JC (1992). Serum carcinoembryonic antigen: a prognostic marker in HIV-related Pneumocystis carinii pneumonia. Scand J Infect Dis.

[10] Chan CM, Chu H, Wang Y (2016). Carcinoembryonic antigen-related cell adhesion molecule 5 is an important surface attachment factor that facilitates entry of middle east respiratory syndrome coronavirus. J Virol.

[11] Fox SE, Akmatbekov A, Harbert JL, Li G, Quincy Brown J, Vander Heide RS (2020). Pulmonary and cardiac pathology in African American patients with COVID-19: an autopsy series from New Orleans. Lancet Respir Med.

[12] Xu K, Chen Y, Yuan J (2020). Factors associated with prolonged viral RNA shedding in patients with coronavirus disease 2019 (COVID-19). Clin Infect Dis.

[13] Tian S, Hu W, Niu L, Liu H, Xu H, Xiao SY (2020). Pulmonary pathology of early-phase 2019 novel coronavirus (COVID-19) pneumonia in two patients with lung cancer. J Thorac Oncol.

[14] Barkauskas CE, Cronce MJ, Rackley CR (2013). Type 2 alveolar cells are stem cells in adult lung. J Clin Invest.

[15] Yu J, Yang Z, Zhou X (2020). Prognostic value of carcinoembryonic antigen on outcome in patients with coronavirus disease 2019. J Infect.

[16] (2022). Clinical Spectrum of SARS-CoV-2 Infection. https://www.covid19treatmentguidelines.nih.gov/overview/clinical-spectrum/.

[17] Wei X, Su J, Yang K (2020). Elevations of serum cancer biomarkers correlate with severity of COVID-19. J Med Virol.

[18] Chang CH, Weng HH, Lin YC, Lin CN, Huang TJ, Chen MY (2023). Association between serum carcinoembryonic antigen and cardiometabolic risks: Implication for cardiometabolic prevention. Front Endocrinol (Lausanne).

[19] Chen Q, Kong H, Qi X (2020). Carcinoembryonic antigen: a potential biomarker to evaluate the severity and prognosis of COVID-19. Front Med (Lausanne).

[20] Dahan S, Segal G, Katz I (2020). Ferritin as a marker of severity in COVID-19 patients: a fatal correlation. Isr Med Assoc J.

[21] Cheng L, Li H, Li L, Liu C, Yan S, Chen H, Li Y (2020). Ferritin in the coronavirus disease 2019 (COVID-19): a systematic review and meta-analysis. J Clin Lab Anal.

[22] Yao Y, Cao J, Wang Q (2020). D-dimer as a biomarker for disease severity and mortality in COVID-19 patients: a case control study. J Intensive Care.

[23] Yu J, Nie L, Wu D (2020). Prognostic value of a clinical biochemistry-based nomogram for coronavirus disease 2019. Front Med (Lausanne).

[24] Lyu P, Liu X, Zhang R, Shi L, Gao J (2020). The performance of chest CT in evaluating the clinical severity of COVID-19 pneumonia: identifying critical cases based on CT characteristics. Invest Radiol.

[25] Yang C, Wang J, Liu J, Huang S, Xiong B (2020). Elevated carcinoembryonic antigen in patients with COVID-19 pneumonia. J Cancer Res Clin Oncol.

